# Noncultured Autologous Adipose-Derived Stem Cells Therapy for Chronic Radiation Injury

**DOI:** 10.4061/2010/532704

**Published:** 2010-12-01

**Authors:** Sadanori Akita, Kozo Akino, Akiyoshi Hirano, Akira Ohtsuru, Shunichi Yamashita

**Affiliations:** ^1^Department of Plastic and Reconstructive Surgery, Nagasaki University Graduate School of Biomedical Sciences, 1-7-1 Sakamoto, Nagasaki 852-8501, Japan; ^2^Takashi Nagai Memorial International Hibakusha Medical Center, Nagasaki University Hospital, Nagasaki 852-8501, Japan; ^3^Atomic Bomb Disease Institute, Nagasaki University School of Medicine, Nagasaki 852-8501, Japan; ^4^Wolrd Health Organization, Geneva 27, Switzerland

## Abstract

Increasing concern on chronic radiation injuries should be treated properly for life-saving improvement of wound management and quality of life. Recently, regenerative surgical modalities should be attempted with the use of noncultured autologous adipose-derived stem cells (ADSCs) with temporal artificial dermis impregnated and sprayed with local angiogenic factor such as basic fibroblast growth factor, and secondary reconstruction can be a candidate for demarcation and saving the donor morbidity. Autologous adipose-derived stem cells, together with angiogenic and mitogenic factor of basic fibroblast growth factor and an artificial dermis, were applied over the excised irradiated skin defect and tested for Patients who were uneventfully healed with minimal donor-site morbidity, which lasts more than 1.5 years.

## 1. Introduction

There is an increasing worry on radiation injuries probably caused by nuclear power plant (NPP) reactor accidents, therapeutic irradiation for malignancy, and interventional radiology (IRV) of unexpectedly prolonged fluoroscopic procedures for cardiovascular diseases such as arrhythmia, ischemic heart diseases, or nuclear medicine of overdose intake of the radioactive for nuclear medicine of internal radiation therapy. The problems are concerning chronic radiation injury as well as how to heal such local and systemic injures acutely. Local chronic radiation injury is resistant to conventional therapeutic modalities such as flap coverage or skin grafting because the deteriorated margins are sometimes indistinguishable from normal intact tissue, and thus sufficient enough debridements are not obtained with surgeons' naked eyes. 

These conditions should be treated properly for the sake of life saving and improvement of local wound healing [1]. However, data of total evidence-based clinical analysis were not established yet. Authors' institute, Nagasaki University, is selected as a global strategic center for radiation health risk control by the Japan's Ministry of Education, Culture, Sports and Technology from FY 2007 to 2011 and exploring to establish such therapeutic regimens, to prevent the radiation injuries, and possibly to regenerate medical and surgical therapy for radiation injuries by using patients' own adipose tissue-derived stem cell therapy. 

Often seen chronic radiation injuries are well handled by sufficient enough blood supply to the radiated tissues, especially in the cartilage, bare bone, and hardened scar tissues. For this purpose, local, distant, and microsurgical vascularized flaps are applied. Recent development of microvasculature of the skin and soft tissues including the connective tissues plays major roles in attributing to accelerate local wound healing. Also, externally administered angiogenic growth factor such as basic fibroblast growth factor (bFGF) together with temporal wound coverage of artificial skin substitute is very effective for those patients with severe injuries, patients with comorbidities, who are intolerant to the extensive and long surgeries [2]. Here, chronic radiation-injured wounds are tested with non-cultured autologous adipose-derived stem cells and clinical implications are discussed.

## 2. Materials and Methods

### 2.1. Treatment of Chronic Local Radiation Injury with Conventional Methods and Stem Cells

Often experienced in radiation therapy for malignancy, cardiovascular modalities should be categorized as difficult wounding with poor vasculature or less healing potentials. 

From January 1990 to April 2007, 10 (8 females and 2 male) patients who demonstrated chronic radiation injuries such as telangiectasia, xerosis, epidermal atrophy karatoses, and fibrosis as well as deep ulcers in the costal ribs and sternum by adjuvant radiation therapy after mastectomy and prolonged fluoroscopic procedures for cardiovascular diseases were surgically treated. 

Other selective clinical cases used angiogenic growth factor namely human recombinant basic fibroblast growth factor (rh-bFGF), which is clinically approved and widely used for clinical wounds in Japan with skin substitutes, which are also clinically available not only in Japan but also in many other nations including USA, the majority of EU nations, and several Asian counties, and the effectiveness of using the artificial skin substitutes in the chronic radiation injuries is temporal coverage and sustainability of both internal and external cells and growth factors. Therefore, combined use of bFGF and artificial skin substitute leads to improved quality of wounds (scar tissue) as well as facilitated wound healing [3]. 

One case was treated with non-cultured autologous adipose-derived stem cell (ADSC) for chronic sacro-coccygeal radiation ulcer in 2008, which was caused by a therapeutic radiation at fractionate 50 Gy at 40 years previously.

### 2.2. Methods

**Figure 1:**
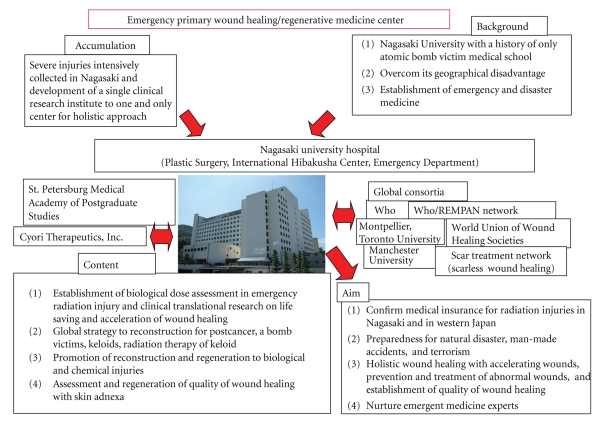
Strategy of emergency radiation injury. Collaborative work with highly established international centers and organ is proposed.

This study was approved by the Ethics Committee of the Nagasaki University Hospital, and written informed consent was obtained from all patients (approved no. 08070296) and partly supported by the Global COE (Center of Excellence) Program E08, Global Strategic Center for Radiation Health Risk Control, and it was funded by the Japan Society for the Promotion of Science. This national research grant enables us to investigate 3 main themes related to radiation health risk: (1) atomic-bomb disease followup cohort research with over 60-year continuous research history, (2) radiation basic science, and (3) international radiation health research. Especially, this radiation regeneration research was involved in further international collaboration framework under international organizations such as WHO (World Health Organization) and IAEA (International Atomic Energy Agency) (Figure 1).

### 2.3. Harvesting of Adipose Tissue by Liposuction and Isolation of ADSCs

3–5 mm incisions, two incisions in the abdomen, four incisions in the thigh, and two incisions in the gluteal region, were made on the abdominal region, the thigh, and the gluteal region. The subcutaneous layer was infiltrated with a lactated Ringer's solution with addition of 0.5 mL of epinephrine and 25 mL of 1% lidocaine per 500 mL. Adipose tissue was suctioned using an 18-G Becker cannula with a 50 mL syringe. Total 250 gram-fat tissues, 120 grams from the abdominal region, 80 grams from the gluteal regions, and 50 grams from the thighs were harvested. 

**Figure 2:**
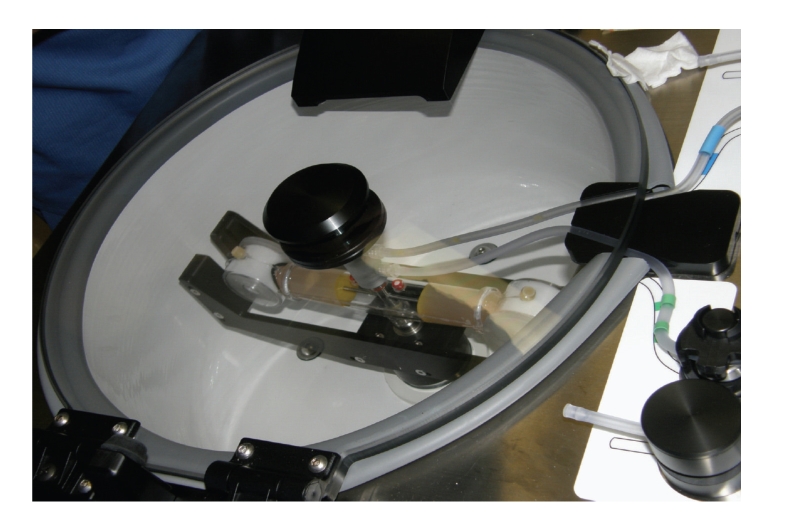
The Adipose-derived stem cells are processed in a closed-circuit machine within 1.5 hours.

 ADSCs were isolated from the suctioned adipose tissue by using the Celution system. (Cytori Therapeutics, Inc., USA). Briefly, the suctioned adipose tissue was introduced into the Celution cell-processing device, which automatically and aseptically extracts and concentrates the mononuclear fraction of adipose tissue and removes unwanted or deleterious cells, cell and matrix fragments such as lipids. By using the Celution system, a 5 mL solution is added to isolated ADSCs in about one and a half hour (Figure 2). The whole procedure is in a closed circuit and this reduces the chance of the contamination.

 The small portion of processed ADSCs was used for the ex vivo cell culture and confirmed the proliferation and differentiation potential. The ADSCs-rich fraction was then plated onto collagen type-I-coated plastic culture flasks in a serum-free medium for primate embryonic stem cells (Primate ES medium, RiproCELL, Tokyo), and the cells, clonally expanded, were collected and stored in Liquid Nitrogen as the primary ADSCs. ADSCs were subcultured when they reached to 80% confluence. Cells were treated with trypsin/EDTA solution, neutralized with tripsin-neutralizing solution, and collected by centrifugation for 5 minutes at 1,200 rpm. The pellects were resuspended in a fresh medium; the number of cells was counted, and 3 × 10^5^ cells were plated into T25 flasks (25 cm^2^) for subculture while the rest of the cells were stored in liquid nitrogen.

### 2.4. Adipose-Derived Stem Cell Grafting and Postoperative Management

**Figure 3:**
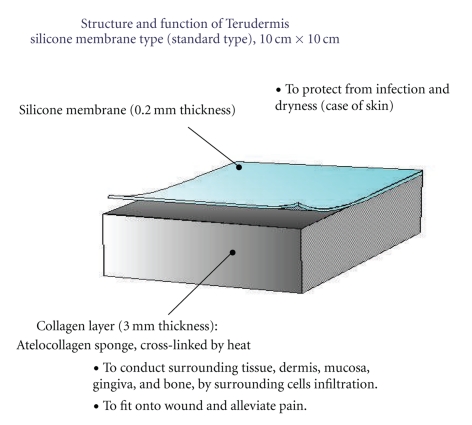
Freeze-dried bilayer artificial dermis made of bovine dermis. The outer membrane of silicone layer is easily removed and easily soaked with cell-containing solution.

For the scaffold purpose, we used the artificial dermis (Terudermis, Olympus-Terumo Biomaterials Co., Ltd., Japan) (Figure 3). The Terudermis is composed of two layers: a lower layer of bovine atelocollagen and an upper layer comprising a silicone sheet which protects against infection and dryness from the outside. After minimum debridement, the Terdermis was multilayered and stacked over freshly debrided wounds. The silicone sheets were removed except top Terudermis. The two-thirds of isolated ADSCs alone were injected; around the debrided wounds, at the base of the wounds, and into Terudermis. Another one-third of ADSCs was mixed with the autologous adipose which was rinsed with a lactated Ringer's solution. In the Celution system, after isolating ADSCs, the disposable cell collection plastic case one was again used to mix the suctioned fat, which is rinsed separately in the 50-cc syringe and repeated until the oil droplets are removed. After being mixed, it was injected into a zone of hard fibrotic tissue around the debrided wounds in 2-cm width in all directions.

### 2.5. Angiogenic Growth Factor and Basic Fibroblast Growth Factor (bFGF)

**Figure 4:**
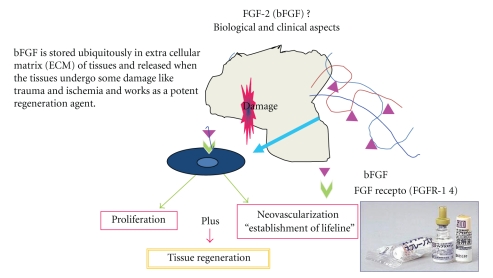
Commercially available growth factor and basic fibroblast growth factor (bFGF). Mode of action is explained and the mechanism is proposed.

Genetically recombinant human bFGF (Fiblast, Trafermin) was purchased from Kaken Pharmaceutical Co., Inc (Tokyo, Japan). The Freeze-dried bFGF was dissolved in 5 mL of benzalkonium chloride containing solution right before the first use and stored at 4°C for one day, with 300 *μ*L sprayed over 30 cm^2^ area from 5 cm distance, and 0.3 mL per day of this solution was applied over the wound. One week after removing the silicone layer, human recombinant fibroblast growth factor (bFGF: Fiblast, Kaken Co., Ltd., Japan) (Figure 4) was sprayed. The wound was covered with nonadherent occlusive foam dressing.

## 3. Results

### 3.1. Treatment of Chronic Local Radiation Injury with Conventional Method

All wounds were healed after several surgical modalities. None of the cases was healed with single procedure (2 to 6 surgeries, mean 4.3). 

Of our cases, one breast-cancer patient was treated by a standardized Halsted method with major and minor pectoralis muscle, radical neck, and axillary and internal mammary lymph node dissections. This patient has undergone 50-Gy fractionate radiation therapy postoperatively. The radiated area showed chest fistula deep to the pleura with surrounding unhealthy hardened scar tissue and chronic inflammation. 

**Figure 5:**
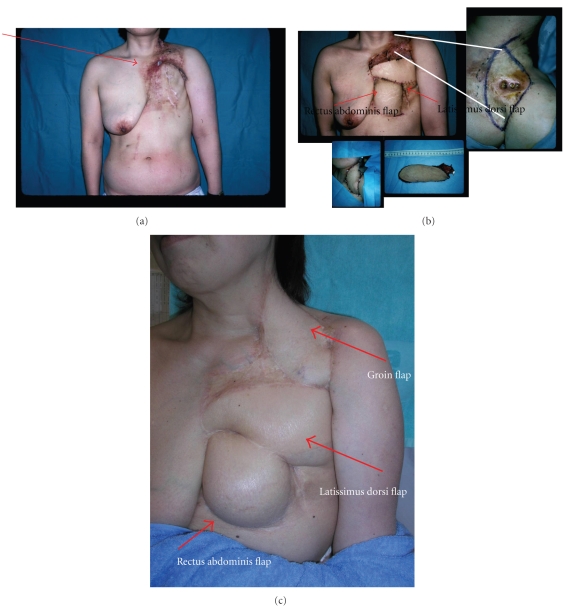
55-year-old woman underwent a left breast cancer surgery by a standardized Halsted methods, followed by 50-Gy fractionate radiation therapy 15 years previously. (a) The chest demonstrates fistula to the costal rib and adjacent to the pleura as the arrow depicts, and the surrounding tissues were firm and various-degree inflammation existed. (b) Sequential three major flaps (rectus abdominis, latissimus dorsi, and free groin flap) are used for total coverage. (c) In 7 years postoperative view. There is irregularity of the scar margins.

The whole affected area was sequentially excised in 3 reconstructive surgeries, starting with rectus abdominis musculocutaneous flap, then latissimus dorsi musculocutanous flap, and finally with groin-free flap. In the course after each surgery, the margin of the flap was partially dehiscent and necrotized, which required further touchups? The total number of the reconstructive surgery was 6 (Figure 5).

### 3.2. Treatment of Chronic Local Radiation Injury with Adipose-Derived Stem Cells

**Figure 6:**
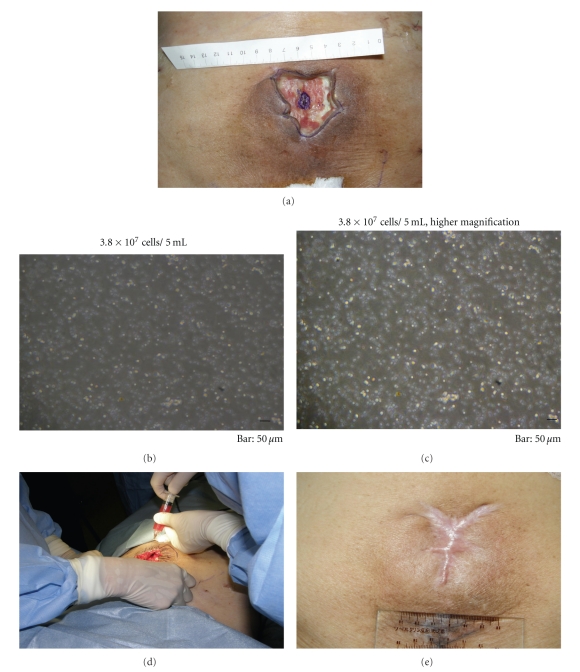
89-year-old woman underwent a uterine cancer surgery followed by 50-Gy fractionate radiation therapy 40 years previously. (a) In 10 × 10 cm area of radiation, 5 × 10 cm area was exposed. Bone, fascia, and muscle as well as skin and fat were affected. (b, c, d) After careful debridement, 3.8 × 10^7^ cells/5 mL were applied over the wound bed and margins and soaked with artificial dermis. In a few days postoperatively, bFGF was sprayed over the peeled-off inner regenerated tissue for 21 days. (e) In 1.5 years postoperative view. The regenerated tissue remained durable, soft, and pliable.

Regeneration method with patient's own non-cultured ADSCs was planned for a patient underwent 50-Gy fractionate radiation therapy for uterine cancer 40 years ago. The pigmented sacrococcygeal region appeared with central intractable wound. Necrotized bone and fascia muscle along with malodour were observed. The ADSCs-treated chronic radiation wounds underwent debridement to remove unhealthy superficial necrotized bone, fascia, periosteum, and muscle. 3.8 × 10^7^ cells in 5-mL of final volume from 250 mL of subcutaneous aspirated fat obtained from nonradiated area were used. Some ADSCs were directly injected in wound bed and margins; others were soaked with the artificial dermis. In a few days postoperatively, the silicone upper layer of the artificial dermis (Terdermis) was removed, and bFGF was sprayed over the regenerated wound for three weeks. There was no significant adverse effect neither in donor site or treated wound. The wound was healed uneventfully by day 82 and no sign of recurrence appeared, but the regenerated tissue developed mature in 1.5 years (Figure 6).

## 4. Discussion

Local radiation injuries caused during medical therapy for malignant tumors [4] and heart disease [5] may be accompanied with systemic symptoms of hematologic, neurologic, and gastrointestinal symptoms such as neutropenia, thrombopenia, fatigability, nausea, and diarrhea by contact to the scrap yard radioactive wastes without notice [6] or exposure to the radiation accidents [7] by touching gammagraphy radioactive source by mistake [8]. Since locally radiated tissues show decreased or insufficient vascularity and tissue damage, demonstrating erythema, teleangiectasia, pigmentation, or dermal atrophy, once wound is developed, it is often intractable and further leading to tissue necrosis, infection, and later fibrosis in demonstrating chronic radiation injury syndrome [9]. Therefore, radiation-injured wounds tend to persist for a long time, show impaired healing, and be prone to recurrence even by minor trauma. Radiated wounds are treated by adequate debridement both in the depth and in the width and covered with well-vascularized tissues or by cultured bone-derived mesenchymal stem cells [8]; however, the long-term outcome is not warranted, and donor-site morbidity and the duration for treatment are sometimes concerned, especially for the aged patients or patients who somehow have problems in harvesting the donors or being limited due to the coexisting diseases. As seen in our reconstructive cases, the surgical modalities constantly required multiple surgeries partly due to the definitive damage-free margins of the affected tissue. 

Application of Stem cell therapies for repair and regeneration has recently been investigated at a clinical level in variously defected or injured tissues, among which stem cells and adipose-derived stem cells (ADSCs) can be harvested with a minimally invasive procedure by liposuction procedure through a small incision. Similar to our method but in detail very different, Clinically purified autologous lipoaspirates were used as treatment for radiotherapy tissue damage of consecutive 20 patients. Indirectly, induced ADRCs have potential in cell therapy for radiation injury due to increasing neovascularization and retention of the fat property [10].

This enables us to adopt this regeneration method for patients with severe comorbidity such as elderly systemic disease and physical wasting state (data not shown). The ADSCs contain several types of stem and regenerative cells, including endothelial and smooth muscle cells and their progenitors and preadipocytes [11]. The ADSCs have the capacity to differentiate into multiple lineages and cell types including mesodermal tissues such as fat, bone, cartilage, endothelial cells of endodermal origin, and neurons and epidermis of ectodermal origin as seen in the mesenchymal stem cells [12]. 

Management of radiation injuries composes two major parts. One is localized injuries and the other is of systemic injuries. Among localized radiation injuries, chronic injuries are more common in the medical field after caner radiation therapy. Usually management of these chronic wounds is well handled by well-vascularized tissue transfers as various plastic surgical procedures have proved. In consideration of each patient general condition and preference, the choice of therapeutic selections should be performed. On the other hand, when the local radiation injuries are encountered in an acute phase, there are high chances for innovative procedures using autologous stem cells. The hMSCs are resistant to radiation. We have previously demonstrated *in vitro* cell proliferation curve and are also able to produce protein avoiding cell apoptosis [13]. And the application of cultured bone-derived mesenchymal stem cells successfully healed severe local radiation wounds. However, the cultured stem cell therapy takes longer period as long as 16 days before cell therapy and required multiple (5 times) cell injections as well as 2 skin grafting, 2 flaps, and 1 artificial dermis coverage [14]. Also, increasing evidences demonstrate that ADSCs are similar to hMSCs in cell properties and characteristics both *in vitro* and *in vivo* [11]. ADSCs are highly yielding and less invasive for donor sites. The acute myocardial infarction porcine models by improving left ventricular function, perfusion, and remodeling [15]. When localized radiation was distant enough from the donor sites adipose tissues, immediate debridement and regeneration happens using adipose-derived stem cells, which are available for processing within 1.5 hours simultaneously in the same operation theater without cell culture since adipose tissues (fat tissues) are abundant in adult humans compared to other stem cell sources. In the limited clinical circumstances of high-risk patients such as elderly and chronic local infection, there is still opportunity of harvesting and processing the patient's own fat-derived stem cells successfully as seen in our case. Practically for emergency radiation injury cases, more abundant cell sources such as fat are the primary candidate for this purpose. The cell property and characterization of ADSCs are discussed and discussed either fresh or cultured [16]. The results from the clinical trial for acute myocardial infarction are expected and may be applicable for acute radiation injury treatment.

For treatment of systemic radiation injuries, stockpiled stem cells should be globally available through medical assistance network system under WHO-REMPAN, in which Nagasaki University is highly involved in its activity, or other international frameworks. Early resurfacing of the damaged skin and subcutaneous tissues is as important as hematological and intestinal system resuscitation [17].

Also, therapeutic guidelines for systemic radiation injuries are anticipated from practical and regulatory view points. Highlighting innovative technology and devices as well as currently existing medicines and devices is expected for the sake of preparing to treat “systemic” radiation injuries most effectively. 

Therapeutic regimens of radiation injuries used to be dependent on each subspecialty in the medical filed such as internal medicine, radiology, and surgery. 

Recent establishment of wound care specialty was mostly led by plastic surgeons, but other supporting specialists such as nurses, dermatologists, and gastrointestinal physicians and surgeons may be practically handling these rare but of significant impact “radiation injuries” as a interdisciplinary approaches. Therefore, more specialization for “radiation injuries” may be required.
